# Faith, Fear, and Facts: A COVID-19 Vaccination Hesitancy Intervention for Black Church Congregations

**DOI:** 10.3390/vaccines10071039

**Published:** 2022-06-28

**Authors:** Bridgette Peteet, Valerie Watts, Eunique Tucker, Paige Brown, Mariam Hanna, Amanda Saddlemire, Miriam Rizk, Juan Carlos Belliard, Jacinda C. Abdul-Mutakabbir, Samuel Casey, Kelvin Simmons

**Affiliations:** 1 Department of Psychology, School of Behavioral Health, Loma Linda University, 11130 Anderson St., Loma Linda, CA 92350, USA; vwatts@students.llu.edu (V.W.); euniquetucker@students.llu.edu (E.T.); pbrown1@students.llu.edu (P.B.); mahanna@students.llu.edu (M.H.); asaddlemire@students.llu.edu (A.S.); mrizk@students.llu.edu (M.R.); 2 Institute for Community Partnerships, Loma Linda University, Loma Linda, CA 92350, USA; jbelliard@llu.edu; 3 Department of Pharmacy Practice, School of Pharmacy, Loma Linda University, Loma Linda, CA 92350, USA; jabdulmutakabbir@llu.edu; 4 Department of Basic Sciences, School of Medicine, Loma Linda University, Loma Linda, CA 92350, USA; 5 Congregations Organized for Prophetic Engagement, San Bernadino, CA 92411, USA; scasey@copesite.org (S.C.); simmons6197@gmail.com (K.S.); 6 Inland Empire of Concerned African American Churches, San Bernardino, CA 92411, USA

**Keywords:** vaccine hesitancy, health belief, COVID-19, medical mistrust, black health disparities, health inequity

## Abstract

Background: Blacks are dying from the novel coronavirus of 2019 (COVID-19) at disproportionate rates and tend to have more COVID-19 vaccine hesitancy than Whites. These disparities may be attributable to health knowledge and government/medical mistrust stemming from negative experiences with the medical system historically and presently (e.g., the Tuskegee Experiment, provider maltreatment). Method: The present study assessed COVID-19 vaccine hesitancy and the effectiveness of a 1.5 h, dialogue-based, web intervention hosted by an academic–community partnership team. The webinar included approximately 220 male and female, English speaking, Black churchgoers in the western U.S. The webinar focused on the psychology of fear and facts about the vaccine development. Results: The sample was mostly females who had higher vaccine hesitancy than men. A third of participants feared hospitalization if they contracted COVID-19. Many participants reported that learning facts about COVID-19 was most impactful. Statistical analyses indicated an increased willingness to get vaccinated after the webinar in comparison to before (t(25) = −3.08, *p* = 0.005). Conclusion: The findings suggest that virtual webinars may be effective at reducing COVID-19 vaccine hesitancy among Black churchgoers and may be applicable in addressing other health behaviors.

## 1. Introduction

The novel coronavirus of 2019 (COVID-19) global pandemic hit the U.S. early in 2020. The virus mortality rate of 5.6% greatly outpaced the seasonal flu that year [1]. This highly transmissible virus quickly spread, prompting statewide stay-at-home orders and mask mandates. Despite these preventative efforts, the risks magnified in minoritized communities.

Essential workers, disproportionately Black and Latinx [2], continued in their high-risk occupations and returned to largely densely populated communities, spreading the virus [3]. The risks of severe symptoms and death were higher for those with certain preexisting health conditions such as asthma, heart disease, and diabetes and racial/ethnic minorities are overrepresented within these disease states [4,5]. As a result, Black, Latinx, and those in low socioeconomic quadrant populations bore the brunt of higher COVID-19 infection rates and subsequent death than Whites [6,7]. National surveillance data found that Blacks were 1.1 times more likely to be infected, 2.3 times more likely to be hospitalized, and between 2 to 3 times more likely to die from complications related to COVID-19 than Whites [8,9].

In December of 2020, the federal Food and Drug Administration approved the emergency use of two COVID-19 vaccines for adults that were more than 90% effective. However, studies indicated that only 49–70% of U.S. residents planned to receive the COVID-19 vaccine, and the rate was lower for Black people at 40% [10]. Vaccine hesitancy is a delay in acceptance or refusal of available vaccines [11]. It tends to be highest among Black people, women, and conservatives [12]. Vaccination status is particularly impactful since those who are vaccinated will experience fewer community and travel restrictions [13]. The World Health Organization’s (WHO) Model of Vaccine Hesitancy indicates that this behavior is attributable to a cluster of three factors (3Cs): confidence, complacency, and convenience [14].

Trust is a key ingredient in vaccine dissemination. Individuals must have confidence that the medication is: (1) safe, (2) delivered by reliable and competent health professionals, and (3) that the motivations of policymakers are transparent [14]. Individuals may feel complacent and underestimate their personal risk of contracting the virus [14]. Vaccines must also be convenient in physical and financial ways to reduce dissemination barriers [14]. The present web-based intervention centered on building vaccine confidence.

In comparison to White people, for Black people the safety of the vaccine was of paramount concern [15,16,17]. In particular, Black people were more uneasy about the vaccine ingredients. Some were apprehensive about the potential for long-term side effects. Others worried about unfounded rumors that the vaccine could change one’s DNA. Confidence in vaccine safety was further compromised by high medical mistrust in Black communities. 

Medical mistrust—or suspiciousness about healthcare scientists and providers—is a major public health concern [18,19]. The majority of U.S. Americans (77%) report low confidence in healthcare [20]. For Black people, historical events such as the Tuskegee Syphilis Study, where hundreds of Black men were left untreated for syphilis, contribute to mistrust even further. Moreover, personal negative experiences in healthcare may also affect trust [19]. Specifically, Blacks and other racial/ethnic minorities receive a poorer quality of healthcare than Whites [21]. Adding in politics further dismantled vaccine confidence. 

The COVID-19 pandemic began under arguably one of the most politically divisive times in U.S. history. The slow, opaque response to the virus from government leadership politicized the vaccine development and dissemination. Black people overwhelmingly tended to side with the opposing political party and as a result they did not trust the process or rapid speed of the vaccine development [19]. Healthcare professionals and government officials need to focus on rebuilding confidence in Black communities to reduce vaccine hesitancy. Experts suggest that at-risk communities need targeted interventions to address their specific concerns [7,22]. 

Dialogue-based interventions are a low-cost strategy to effectively reduce vaccine hesitancy [23]. The World Health Organization (WHO) recommends that interventions should: (1) clearly identify a target audience, (2) facilitate meaningful engagement, (3) consider contextual and multi-level influences, (4) address population-specific concerns in a multifaceted manner, and (5) evaluate the effectiveness [13].

Interventions using community–academic partnerships (CAP), “involving community stakeholders as partners in research” [24], may be one effective way to deliver these messages to vaccine hesitant populations. More than 83% of Black people believe in God [25]. Thus, connecting with the faith community is a useful strategy to reach and engage with Black people to provide mental health interventions while keeping social context in mind [26]. Black faith leaders are often the ear of the community and can help identify specific concerns. The quality of community interventions varies and even promising interventions may not be evaluated or published [27]. 

The disproportionate gap in COVD-19-related health conditions and vaccine hesitancy in minoritized communities is an urgent public health issue. Some well-meaning community agencies are scrambling to intervene without the benefit of evidence-based strategies while health institutions neglect to rebuild community trust prior to providing health education. To date, there are no identifiable data-driven, targeted community interventions to decrease COVID-19 vaccine hesitancy among Black people. The present study will help address this gap. 

The present pilot intervention, titled the COVID-19 Faith Summit intervention, had four main objectives:

1. Assess COVID-19 vaccination hesitancy within the Black churchgoing community.
2. Facilitate an intervention designed to quell fears and provide factual information about the COVID-19 vaccine development with a panel of experts.
3. Determine the effectiveness of the intervention.
4. Disseminate the intervention to a broader audience.

## 2. Methods

The COVID-19 Faith Summit used the aforementioned evidence-based strategies to conduct a vaccine hesitancy webinar using a community–academic partnership to target Black faith communities. The findings may have useful implications in determining the applicability of existing vaccine hesitancy interventions for use with COVID-19 and within minoritized communities. The approach may be a useful model for rebuilding community trust in healthcare and closing the COVID-19 health gap in Black communities. 

The CAP team included two Black church community organizations and a local university. The leaders of the two church organizations represent a collective of more than 30 area congregations. The university is a faith-based health sciences educational institution. Two senior faculty members, one from public health and the other from psychology, served as the principal investigators on the research component. Additionally, two doctoral research assistants assisted with the pilot project. All members of the CAP worked collaboratively to identify the objectives; design, implement, and evaluate the intervention; and disseminate the results. 

Members of the faith community reached out to academic partners from prior projects and invited them to discuss vaccine fears and facts with churchgoers and community members affiliated with their respective congregations, and the CAP team (described above) was formed. Members of the CAP team reflected the race/ethnicity (i.e., Black, Biracial) and gender of the target audience for the vaccine hesitancy webinar. 

The CAP team identified additional expert panelists including a researcher involved in the development of one of the authorized COVID-19 vaccines, the university president affiliated with the academic members of the CAP team, and a county public health officer. All additional expert panelists held doctorate degrees in medicine.

The church leaders of the CAP team facilitated the panel. The academic members of the CAP team served on the panel as experts in their respective fields. Lastly, the additional expert panelists presented COVID-19 information relevant to their respective positions.

Members of the CAP team conducted informal inquiries through social media and personal and professional networks to identify the specific fears circulating about the COVID-19 vaccine within the Black community. The themes of the responses centered on: government/historical mistrust, timeline of the vaccine development, and potential side effects. Generally, women seemed to exhibit more vaccine hesitancy than men did. Men seemed to report having less fear of the vaccine and a sense of urgency to return to normalcy. Overall, this information was used to complement the literature on vaccine hesitancy among Black people and to inform the webinar subtopics. 

The CAP team held two planning meetings in advance of the webinar. During the first meeting, the team members shared information about fears and misinformation about the vaccine that emerged from their varied networks. Based on this information, during the second meeting the team drafted a 1.5 h webinar agenda, which included three primary segments: (1) the importance of the vaccine, (2) the psychology of fear, and (3) facts about the immunization. 

The community team representatives set up the technical mechanism for the Zoom webinar. Advertising was done between 5 and 7 days in advance of the webinar and posted on congregation websites, Flocknote mass emailer, social media, and through digital church announcements. In addition, members of the partnership team and invitees distributed the webinar through their affiliated networks at the institution and area churches by email. In total, there were approximately 220 participants in the virtual webinar, excluding panelists. The vast majority of participants appeared to be Black women.

The academic team representatives obtained exemption from the institutional review board (IRB) at their affiliated institution to conduct the present study. 

The webinar occurred in December of 2020. This was approximately 10 months after COVID-19 began to restrict the work and social activities of U.S. residents and marked the beginning of vaccine rollouts. 

One of the community faith leaders opened the webinar with a prayer based in the Christian tradition. The other community faith leader reviewed webinar etiquette and introduced members of the expert panel. A member from the academic team read a brief research consent script at the start of the webinar. Participants were encouraged to follow the link posted in the chat that directed them to a survey. The research assistants sent two reminders in the chat box to encourage participants to complete the baseline survey. During the last 10 min of the webinar, participants were asked to complete the follow-up survey and were sent reminders via chat as the webinar neared its conclusion. 

The homepage of the Qualtrics survey included a detailed consent to participate. Participants indicated consent by clicking the affirmative button. Each survey took less than two minutes to complete. No identifiers were collected on the participants. 

During the session, webinar participants were invited to use the Zoom chat feature to ask questions. Panelists who were not presenting at that particular time responded to inquiries with empathy and as appropriate to their expertise. 

During the webinar, the two faith leaders alternated asking the panel members pre-vetted questions based on the discussions in the planning meeting. The global health expert (academic partner) gave brief opening statements about COVID-19 health disparities, highlighted the benefit of faith and science intermingling, and provided inspirational images including the first U.S. person to receive the vaccine (i.e., a Black nurse). A licensed clinical psychologist (academic partner) facilitated the following: (1) informed consent for study participation; (2) brief historical information on medical mistrust and provider maltreatment; (3) an overview of modern research ethics; (4) a discussion of the psychology of fear (e.g., fight, flight, freeze, face model) and strategies to overcome fear and make informed rational choices related to the vaccine. 

The invited medical expert from the Moderna vaccine clinical trials then discussed the specifics of the process, timeline, and development of COVID vaccines. He emphasized that the vaccine was developed based on existing science and that there were no shortcuts in its development but rather to reduce bureaucratic barriers. 

To emphasize institutional support for community partnerships, the university president of the affiliated institution gave remarks about the safety of the vaccine and made a commitment for ongoing CAPs. The county representative then discussed plans for a regional roll out. Each panelist had a final opportunity to provide a closing remark. Finally, a guest pastor closed the program with another prayer also in the Christian faith tradition. The psychologist and guest pastor were women, and all other panelists were men. 

The academic team members drafted survey questions based on the Pew Research Center COVID Attitudes Survey [10]. The community team members vetted and approved the survey items. The electronic Qualtrics survey was confidential and no identifying information was collected. 

The baseline survey included four questions, two demographic (i.e., age, gender) and two vaccine-related items (i.e., concern about contracting COVID, vaccine hesitancy). The post-survey consisted of four items asking whether they personally know someone with COVID, whether the presentation helped them to think more about the vaccine, about the most helpful information from the presentation, and about vaccine hesitancy (duplicated from the pre-survey; see Table 1 for survey items). 

The recorded webinar was posted on social media pages (i.e., Facebook, Instagram) associated with CAP members. The Facebook webinar video has 268 views to date. A member of the press provided news coverage of the event on a locally affiliated station of the national public radio (NPR; see https://www.kvcrnews.org/post/local-african-american-faith-community-hosts-virtual-conversation-vaccine; accessed on 1 January 2021). The preliminary and final survey findings were shared with all members of the CAP for broader dissemination to the target community.

## 3. Results

Data were downloaded from the online database, Qualtrics, into SPSS version 27. The data were cleaned by removing any participants who were under the age of 18 and/or did not identify as Black or Latinx, as well as those who did not complete more than 75% of the questionnaire. A descriptive analysis was then computed. 

Cross-sectional main analysis was used to assess the associations between vaccine hesitancy at baseline and follow-up as well as age and gender differences among webinar participants.

Nearly half (47.2%) of the 225 webinar participants completed the baseline survey. One-fifth of webinar participants (*n* = 44) completed the follow-up survey. The age distribution of participants was varied and the majority were between 30 and 49 years of age (see Table 2). Most participants identified as female (*n* = 69). The overwhelming majority of participants (88.1%) who responded to the survey reported that they personally knew someone who had been diagnosed with COVID-19. Over a third (38.6%) endorsed concerns about requiring hospitalization if they should contract the virus. 

The COVID-19 vaccine “hesitancy” variable was reverse coded, such that a higher score on the variable indicated more positive views towards getting the vaccine (e.g., Definitely Yes = 5; Definitely No = 1). Results from the baseline survey indicated that 39.8% of participants definitely or probably would get the COVID-19 vaccine. The post-survey yielded about a 12% increase (52.4%) on this item. The mean scores on baseline vaccine willingness suggest neutral attitudes (*M* = 2.88, *SD* = 1.37) and then somewhat positive willingness after completing the webinar (*M* = 3.27, *SD* = 1.37). Responses from participants who completed both the baseline and follow-up surveys were compared using a paired-samples *t*-test (*n* = 26). The paired samples *t*-test showed an increased willingness and reflected a significant change *t*(25) = −3.08, *p* = 0.005 (see Table 3). 

To assess demographic differences, both the gender and age variables were recoded into dichotomous variables. The two participants who identified as “Other/Prefer not to answer” were removed from the analyses. The age variable was recoded as participants 18–49 years old and those 50–65+ years old. This was done to balance the sample size and assess potential generational differences. Independent sample *t*-tests found a significant mean difference in vaccine hesitancy between male (*M* = 3.88, *SD* = 1.41) and female (*M* = 2.90, *SD* = 1.32) participants prior to the COVID-19 webinar, *t*(84) = 2.72, *p* < 0.01. Such that, at the start of the COVID-19 webinar, male participants had more positive vaccine willingness than females. There were no significant mean differences between male and female participants’ vaccine hesitancy after the webinar, *p* > 0.05. There also were no significant mean differences in vaccine hesitancy between age groups prior to or post the COVID-19 webinar, *p* > 0.05 (see Table 4).

Of the responses about the most influential aspects of the webinar (*n* = 36), participants (44.4%) were most interested in vaccine facts (e.g., explanation of ingredients, side effects, clinical trial results, and MRNA mechanisms) and others (30.6%) found the expert panelist presentations most meaningful.

## 4. Discussion

Ethnic and racial minority communities have 10–50% higher COVID-19 mortality risk when compared to Whites in the U.S. and the UK [13]. Blacks were less likely to participate in the COVID-19 vaccine clinical trials and have high rates of vaccine hesitancy [10,27]. Barriers to participation include distrust of the medical community, health disparities in access and quality of healthcare, a lack of recruitment, inadequate awareness, and language and cultural barriers [28]. The aim of the COVID-19 Faith Summit pilot intervention was to assess baseline vaccine hesitancy, pilot an evidence-informed health education webinar, determine participant attitude change regarding the COVID-19 vaccine, and disseminate the intervention among Black churchgoers. The present study found that collaborative CAPs may be useful at reaching the Black community and reducing vaccine hesitancy within this high-risk group.

Contextualizing the results using components of the Health Belief Model [29], we found evidence to suggest that modifying factors (e.g., race/ethnicity, gender, and age) and changes in individual perceptions of susceptibility, seriousness, and benefits may have influenced participants’ perceived likelihood of action towards vaccine uptake. 

The informal inquiry and data analysis found that Black males had more vaccine willingness than females. This is consistent with national data, which suggest that women are more reluctant to get the vaccine [12]. The study found no age differences, suggesting equivalent vaccine hesitancy across generations. However, researchers should continue to assess generational differences. Younger adults are more likely to take risks and may join the vaccine uptake more rapidly than older adults who have a higher need for vaccine protection [30]. 

Participants reported that the most influential aspects of the webinar were the expert panel and hearing from a Black physician researcher involved in the development of the vaccine. This is in line with prior research that suggests that (1) the ability to identify with the speaker and (2) the perceived competence of a presenter have a strong impact on the persuasiveness of presentations [31,32].

Mortality salience, or reminders of one’s own mortality (e.g., death toll reported, masked social distancing, knowing someone with COVID-19), can increase the personal salience and perception of risk [33]. However, while the vast majority of participants personally knew someone who had contracted COVID-19, few participants were concerned about requiring hospitalization if they contracted the virus. These findings may be particularly problematic due to the intermittent, surge-related shortages of hospital beds and respirators (e.g., Southern California region [34]). Treatment inaccessibility exacerbates mortality risks. Disseminating broad, race-specific mortality data may be necessary to achieve the expected effects of mortality salience. 

The intervention reached a large group with the assistance of videoconferencing technology. Social interactions are restricted during the pandemic and the use of virtual tools is an excellent strategy to reach large audiences; however, face-to-face interventions could have an even stronger participant impact. The 90 min duration of the webinar is best practice to deliver the content and sustain attention [35]. The intervention used an evidence-informed design rather than structure from opinion; recognized historical, personal, and political fears about the vaccine; and used psychological information about decision making to prime participants towards openness to new information. The panelists were cognizant to limit discussing conspiracy theories, which can undermine health education interventions. Instead, misinformation was addressed with empathy, disclosure, and accurate information [23]. 

Another strength was that several members of the CAP team have longstanding relationships, which conveys trustworthiness and engagement with the target community and is consistent with gold standards in community research [36]. Incorporating cultural traditions (i.e., faith) into the intervention was also a beneficial strategy that aligns with best practices in research with the Black community [37]. 

Limitations of the present study include response rates and generalizability. Nearly half of the webinar participants completed the baseline survey. This represents a strong response rate since Blacks are less likely than Whites to respond to surveys [37,38]. We are unable to infer anything about the webinar attendees who did not respond to the survey. Perhaps only those with strong opinions participated in the study. It is also possible that the 90 min length of the webinar was a limitation. Many factors could have contributed to each individual’s ability to stay for the full webinar, such as motivation level, lack of time, previous commitments, and more. It is difficult to infer which factors specifically were relevant barriers at this time. The response rate also dropped to 20% at follow-up, which is more in line with typical survey response rates [38] but suggests that study retention needs to be considered in future interventions. The imbalanced baseline and follow-up survey response rates also impeded the ability to compare changes in individual scores rather than group scores. 

The webinar focused on specific fears identified in the Black community in Southern California. Thus, it may not be generalizable to other races/ethnicities or in other regions. The majority of the survey participants were female; however, this is consistent with the gender representation of Black churches in the U.S. [39]. Black men are at high risk for COVID death and targeted recruitment is needed in follow-up interventions. Future replications of this intervention should aim to address some of these limitations.

Other limitations include the use of virtual webinar platforms and a possible lack of technological literacy among participants. This limitation may have also impacted the accessibility of registration and the ability to attend the webinar. The method of collecting and identifying the fears of the Black churchgoers may not be representative of the Black community as a whole. Further, attitude change around vaccine hesitancy does not always translate into actually receiving a vaccination.

## 5. Conclusions

The COVID-19 Faith Summit was hosted during the first few days of the vaccine distribution, making the need to address vaccine hesitancy extremely timely and relevant. Regional efforts coupled with new federal activities to rebuild trust and provide science-based recommendations should help to reduce vaccine hesitancy among Black people. Scientists and practitioners need to accept responsibility for past atrocities and strive to eliminate current injustices (e.g., high medical errors, lower quality of care [40]) and work to build trust with these communities before conducting health education interventions. Well-intentioned social agencies and organizations have mobilized to reduce vaccine hesitancy through dialogue-based interventions within the Black community. More robust teams which include both church leaders and members of the target population (e.g., adult church members [41] may enhance the design and reach of the interventions. 

### Practical Implications

Intervention methods should be designed and delivered using the best available scientific evidence [38] and academic partners may be useful partners in these efforts. Evaluating and disseminating the outcomes of these interventions provides additional evidence for effective strategies to address vaccine hesitancy. Vaccine uptake is increasingly important given the freedoms vaccine card holders may receive [13]. 

Black people face disproportionately negative health and mortality outcomes related to COVID-19, yet a large portion of this group expresses vaccine hesitancy. The findings of this study indicate that faith-based CAPs are effective in reaching large audiences, designing a targeted and influential health webinar, and increasing willingness to get the vaccine. Broader dissemination of evidence-based interventions such as the COVID-19 Faith Summit will help close the gap on COVID-19 health inequities by increasing vaccine uptake within historically marginalized communities.

## Figures and Tables

**Table 1:** Pre- and post-survey items.

Pre-Webinar Survey	
Item	Response Option
What is your age?	18–29 30–49 50–64 65+
What is your gender?	Male Female Other/Prefer not to answer
I am concerned that I well get COVID and require hospitalization.	Yes No Not sure
If the COVID-19 vaccine is available today, I would get it.	Definitely YES Probably yes Unsure Probably no Definitely NO
Post-Webinar Survey	
I personally know someone who has had COVID-19.	Yes No Not sure
This presentation has helped me to think more about the COVID-19 vaccine.	Yes No Not sure
The most helpful information for today’s presentation was:	[Open text] Not sure
If the COVID-19 vaccine is available today, I would get it.	Definitely YES Probably yes Unsure Probably no Definitely NO

**Table 2:** Demographics of baseline participants (*n* = 104).

	*n* (%)
Age	
18–29	6 (5.8)
30–49	36 (34.6)
50–64	33 (31.7)
65+	13 (12.5)
Missing	16 (15.4)
Gender	
Female	69 (66.3)
Male	17 (16.3)
* Other	2 (1.9)
Missing	16 (15.4)

* Note: Other = Other/Prefer not to answer.

**Table 3:** Mean differences in paired samples of COVID-19 vaccine hesitancy.

*n*	*M*(*SD*)	*t*(*df*)	95% CI	*p*-Value	*d*
		−3.08 (25)	(−0.64, −0.13)	<0.01	0.61
26	2.88 (1.37)				
26	3.27 (1.37)				

**Table 4:** Differences by demographic groups in baseline and follow-up COVID-19 vaccine hesitancy.

	*n*	*M*(*SD*)	*t*(*df*)	95% CI	*p*-Value	*d*
Baseline						
Gender			2.72 (84)	(0.26, 1.70)	*p* < 0.01	0.72
Male	69	3.88 (1.41)				
Female	17	2.90 (1.32)				
Age			−1.75 (86)	(−1.10, 0.07)	*p* > 0.05	0.38
18–49	42	2.83 (1.36)				
50–65+	46	3.35 (1.39)				
Follow-Up						
Gender			0.34 (23)	(−1.35, 1.87)	*p* > 0.05	0.17
Male	3	3.50 (1.73)				
Female	37	3.24 (1.37)				
Age			−0.19 (24)	(−1.28, 1.05)	*p* > 0.05	0.08
18–49	27	3.20 (1.14)				
50–65+	13	3.31 (1.54)				

## Data Availability

The data that support the findings of this study are available from the corresponding author, B.P., upon reasonable request.
